# Freeze–Thaw Behavior and Degradation Modeling of Shale-Based Lightweight Structural Concrete

**DOI:** 10.3390/ma19071361

**Published:** 2026-03-30

**Authors:** Shijie Liao, Jianxin Peng, Guohui Cao, Zhiren Tao, Xu Zhou, Li Dai

**Affiliations:** 1 School of Civil and Environmental Engineering, Changsha University of Science and Technology, Changsha 410114, China; csliaoshijie@163.com (S.L.);; 2 School of Civil Engineering, Hunan City University, Yiyang 413000, China; 3 CCCC Road & Bridge South China Co., Ltd., Zhongshan 528400, China; 4 Jiangxi Communications Investment Maintenance Technology Group Co., Ltd., Nanchang 330200, China

**Keywords:** lightweight structural concrete, freeze–thaw cycles, compressive strength degradation, gamma process, durability modeling

## Abstract

**Highlights:**

**What are the main findings?**

- Freeze–thaw deterioration patterns of LSC were experimentally characterized.
- A Gamma process-based stochastic model was developed for compressive strength degradation under freeze–thaw cycles.
- The degradation mechanism of LSC under freeze–thaw cycles was clarified.

**What are the implications of the main findings?**

- The identified degradation evolution patterns provide quantitative reference data for durability evaluation and material selection of LSC in cold-region engineering.
- The Gamma process-based model allows probabilistic prediction of long-term strength deterioration, offering a theoretical basis for reliability-oriented durability design.
- The clarified degradation mechanism improves understanding of freeze–thaw damage in lightweight aggregate concrete, guiding optimized mix design for enhanced frost resistance.

**Abstract:**

The freeze–thaw cycles lead to cumulative damage and gradual strength deterioration in concrete, which cannot be accurately represented by traditional empirical models. To address this issue, shale-based lightweight structural concrete (LSC) specimens with four strength grades (LSC20-LSC50) were subjected to basic mechanical performance and freeze–thaw cycle experiments. The study investigated the patterns of mass loss, relative dynamic elastic modulus, and loss of compressive strength in LSC subjected to varying numbers of freeze–thaw cycles. Furthermore, the correlation between the dynamic modulus of elasticity and compressive strength was examined. A Gamma process-based stochastic degradation model for the compressive strength of LSC was then developed. The results show that the compressive strength degradation of LSC under freeze–thaw cycles follows a monotonically increasing trend that gradually stabilizes, with low-strength LSC deteriorating faster than high-strength LSC. After 200 cycles, the compressive strength degradation of LSC30 and LSC50 was only 32.66% and 29.79% of that of their corresponding ordinary concretes (C30 and C50). The proposed Gamma process model showed high fitting accuracy for all strength grades of LSC (R^2^ > 0.96, RMSE < 0.25 MPa, MAPE < 11%). The research results provide a scientific basis for the structural design of concrete in cold regions.

## 1. Introduction

In cold regions like Northern Europe, Canada, and Northern China, freeze–thaw damage significantly compromises the durability of concrete structures. Repeated freeze–thaw cycles not only degrade the mechanical properties of concrete but also accelerate crack propagation and internal damage, substantially reducing its service life [1,2,3,4]. According to statistics, the global annual cost of repairing and replacing concrete infrastructure due to freeze–thaw damage amounts to billions of dollars [5]. Against this backdrop, shale-based lightweight structural concrete (LSC), characterized by its low density, relatively high strength, and favorable thermal insulation, has attracted increasing attention for engineering applications in cold regions [6,7,8,9,10]. Compared to conventional concrete, the porous structure of LSC can alleviate the internal pressure caused by pore water expansion during freeze–thaw cycles, thereby enhancing its freeze–thaw resistance [11,12]. However, the long-term durability of LSC in harsh environments still faces considerable challenges, and its freeze–thaw damage mechanisms and performance degradation patterns are not yet fully understood. Therefore, conducting in-depth research on the strength degradation of LSC under freeze–thaw cycles is of significant theoretical and practical value.

Over the past decade, substantial experimental and theoretical studies have been conducted globally on the performance degradation of LSC under freeze–thaw cycles. For example, Kucharczyková et al. [13] investigated the influence of freeze–thaw cycles on lightweight concrete and showed that repeated freezing and thawing affected not only compressive and splitting tensile strengths, but also fracture-related parameters such as fracture toughness and fracture energy. Polat et al. [14] demonstrated that moderate use of lightweight aggregates and air-entraining agents enhances concrete frost resistance by regulating pore structure, while excessive replacement compromises freeze–thaw performance because of reduced strength and higher water absorption. Zeng et al. [15] found that the addition of polypropylene and basalt fibers can significantly enhance the freeze–thaw resistance of LSC and proposed a concrete strength prediction model that takes into account size effects and load types. Kucharczíková et al. [16] showed that the freeze–thaw resistance of LSC is significantly affected by the pre-soaking condition of porous aggregates through its influence on moisture distribution, pore structure, and the interfacial transition zone, with proper pre-conditioning mitigating freeze–thaw damage and unfavorable saturation accelerating durability deterioration. Rustamov et al. [17] examined the freeze–thaw-induced mechanical degradation of fiber-reinforced LSC under up to 300 cycles, showing that steel and PVA fibers improve strength retention and crack resistance. Gan et al. [18] conducted experimental studies to examine how freeze–thaw damage and cyclic loading affect the pore structure of concrete, providing a quantitative analysis of the correlation between the pore fractal dimension, strength, and elastic modulus of the material. Most existing studies primarily evaluate freeze–thaw performance using macroscopic mechanical indicators such as mass loss rate, relative dynamic elastic modulus, and compressive strength. However, the evolution of the internal structure and the underlying micro-damage mechanisms during freeze–thaw cycling have not been fully clarified, making it difficult to reveal the intrinsic degradation behavior of LSC under freeze–thaw conditions. Moreover, most studies have focused on a single strength grade and lack a comprehensive investigation into the performance differences and mechanical damage evolution of LSC with varying strength grades.

Concrete performance degradation under freeze–thaw environments is a complex and progressive process involving not only mass loss and dynamic elastic modulus reduction, but also the cumulative effects of repeated freeze–thaw cycles and changes in internal structure [19]. Researchers have conducted studies based on empirical models and physical mechanism models to quantify the cumulative freeze–thaw effects on performance degradation. For example, Qiu et al. [20] developed a freeze–thaw deterioration model for coal gangue concrete and analyzed its mechanical behavior and stress–strain relationship through uniaxial compression and acoustic emission tests, constructing a constitutive relationship incorporating freeze–thaw and load damage. Yan et al. [21] analyzed the effects of freeze–thaw cycles on the ultrasonic wave velocity attenuation characteristics and dynamic elastic modulus of concrete with three types of inorganic coatings and developed an equivalent freeze–thaw damage model for ultrasonic wave velocity in concrete. Dai et al. [22] linked laboratory-accelerated testing with the on-site service life of concrete, adopting a 60% reduction in relative dynamic elastic modulus as the freeze–thaw failure criterion and formulating a service life prediction model based on the rapid coefficient method. Lv et al. [23] studied the freeze–thaw degradation of diatomite-modified concrete and developed a compressive-strength evolution model based on the relative dynamic elastic modulus, together with a Wiener-process-based life prediction method. Zhou et al. [24] analyzed freeze–thaw damage through damage velocity, accelerated damage, failure, and reliability life curves, developing a freeze–thaw damage model using the Weibull probability distribution. Zhao et al. [25] used the parabolic model, natural attenuation model, and two-parameter Weibull distribution model to fit the evolution of the relative dynamic elastic modulus of recycled reed straw brick aggregate concrete. Results showed that the parabolic and two-parameter Weibull distribution models provided predictions closely matching experimental results, with fitting rates exceeding 90%. Although cumulative freeze–thaw deterioration has been considered in previous studies, most existing models are still mainly based on the relative dynamic elastic modulus and limited freeze–thaw test data. In this context, incorporating compressive strength into freeze–thaw damage modeling can complement conventional stiffness-based evaluation and provide a more complete description of the progressive deterioration of concrete under repeated freeze–thaw actions. Therefore, establishing a probabilistic model for the cumulative degradation of compressive strength is important for improving freeze–thaw damage assessment and supporting more reliable long-term durability evaluation.

This study designed four strength grades of LSC (LSC20-LSC50) and carried out tests on the basic mechanical properties and freeze–thaw resistance. The mass, compressive strength, and relative dynamic elastic modulus loss were systematically analyzed across different freeze–thaw cycles. The freeze–thaw damage evolution and degradation mechanism of LSC were further elucidated based on experimental data. Furthermore, the correlation between compressive strength and dynamic elastic modulus was explored. Compared with previous studies mainly based on empirical deterioration laws, this work introduces a Gamma process-based stochastic framework to characterize the cumulative compressive strength degradation of LSC under freeze–thaw cycles, providing a probabilistic approach for durability prediction.

## 2. Experimental Program

### 2.1. Concrete Materials and Composition

**Figure 1:**
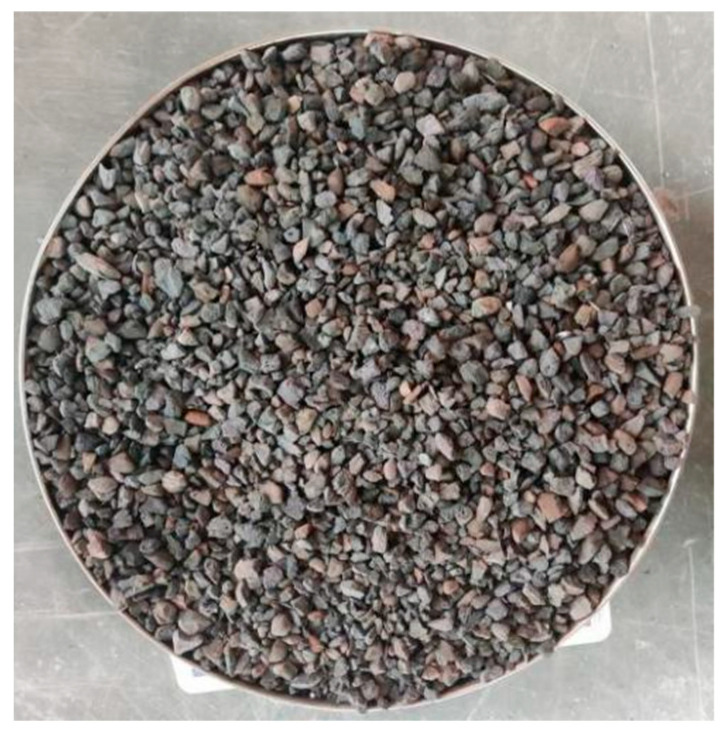
Appearance of shale ceramic.

**Figure 2:**
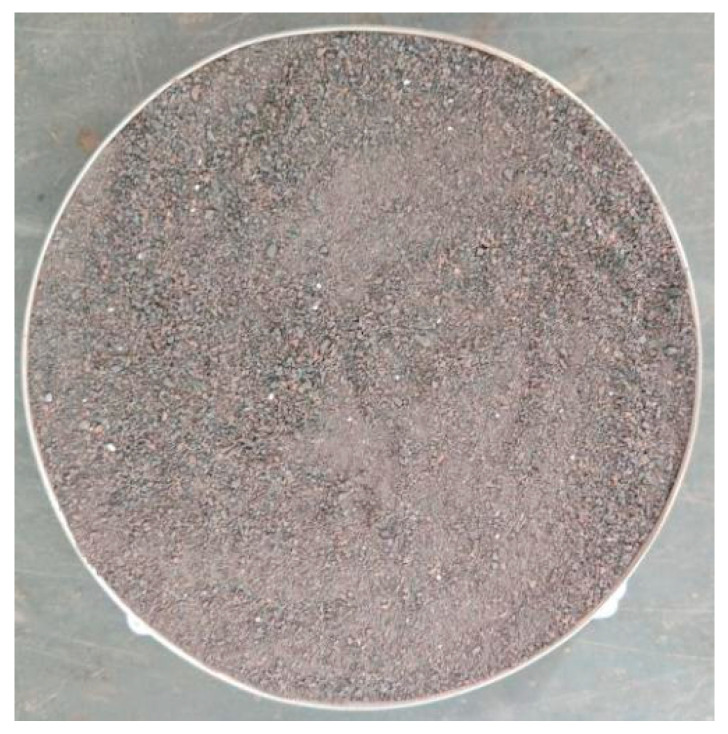
Appearance of shale pottery sand.

**Figure 3:**
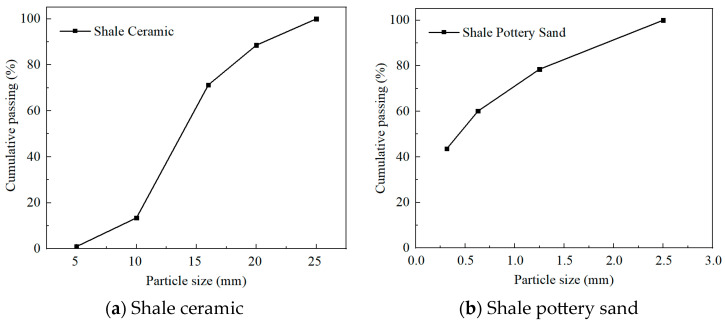
Particle size distribution of aggregates.

LSC consisted of ordinary Portland cement, urban tap water, continuously graded 4.50–20.00 mm shale ceramic particles, 0.00~1.25 mm shale ceramic sand, ball milling powder, and polycarboxylate water reducer. Four strength grades of LSC—LSC20, LSC30, LSC40, and LSC50—were designed in this test. The major chemical compositions of the materials are summarized in Table 1, and the LSC mix ratios were presented in Table 2. Among them, LSC20 and LSC30 specimens are made of P.C 42.5 common Portland cement, while LSC40 and LSC50 use P.O 52.5 common Portland cement. The shale ceramic (Figure 1) and shale pottery sand (Figure 2) used in this study were manufactured by Hunan Huaxin ceramic Particle Technology Co., Ltd., (China, Yiyang) and their particle size distributions were shown in Figure 3.

### 2.2. Preparation of Specimens

**Figure 4:**
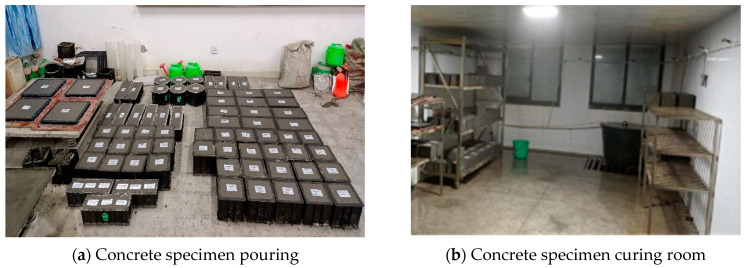
Specimen preparation.

The specimens were grouped into four categories based on strength grade: LSC20, LSC30, LSC40, and LSC50, with each group consisting of ten specimens measuring 100 × 100 × 400 mm for freeze–thaw tests. In addition, 160 cube specimens of 150 × 150 × 150 mm and 160 prismatic specimens of 150 × 150 × 300 mm were made for cube compression, axial compression, and elastic modulus tests, as shown in Figure 4. The specimens were cured in a standard curing room (20 ± 2 °C, RH > 95%) for 28 days before testing.

### 2.3. Mechanical Properties

It is important to carry out basic mechanical properties tests before conducting freeze–thaw cycle experiments. In this paper, the cubic compressive strength, axial compressive strength, and elastic modulus tests of LSC are measured according to the standard for test methods of concrete physical and mechanical properties (GB/T 50081-2019) [26]. The tests were conducted using a YA-2000C hydraulic compression testing machine with a maximum load capacity of 2000 kN. The test results are summarized in Table 3. To further verify the dispersion of the test data and evaluate the representativeness of the mean values, the statistical parameters of the 28-day cube compressive strength of LSC with different strength grades were calculated, including the standard deviation and coefficient of variation (see Table 4). The results show that the coefficients of variation for all specimen groups are less than 3%, indicating low data dispersion and minor differences among specimens, thus confirming good stability of the experimental results.

### 2.4. Testing Methods

**Figure 5:**
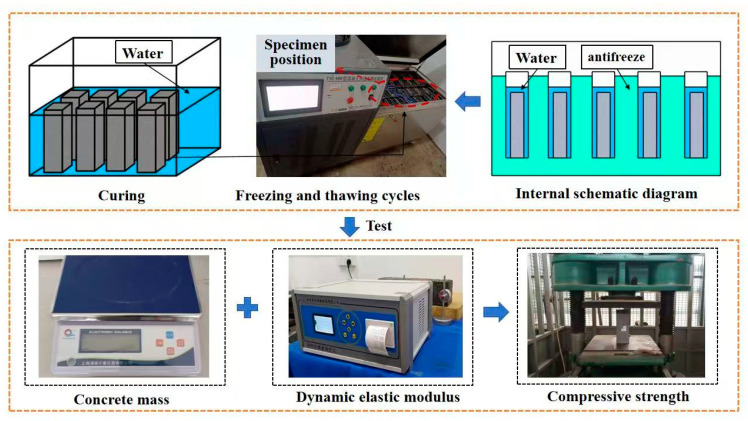
Freeze–thaw test flow.

There are many testing approaches for concrete freeze–thaw cycles, for instance, slow freezing, rapid freezing, the single-sided freeze–thaw method, etc. Among them, the rapid freezing method has good reliability and correlation with the actual freeze–thaw environment [27]. Therefore, this study adopted the rapid freezing method in accordance with ASTM C666/C666M-15 [28], and the testing process was presented in Figure 5. When the concrete specimen reaches the age of 24 days, soak the specimen in clean water for 4 days. After saturation, the sample was taken out and dried to eliminate surface moisture, followed by measuring the initial mass and the initial dynamic elastic modulus. Each specimen was measured three times, and the average value was recorded. After the test was completed, the specimen was placed in the rapid freeze–thaw apparatus, and tap water was added to the specimen box to cover the top of the specimen by about 5 mm, then the freeze–thaw cycle test was started. Each cycle lasted for 4 h, with a temperature range of −18 °C to 5 °C. In this study, 200 freeze–thaw cycles were adopted to evaluate the frost resistance of concrete. This complies with the upper limit requirement of ASTM C666/C666M-15, ensuring representative results and practical comparability [29,30]. During the first 100 cycles, the specimens were tested every 25 cycles (including visual inspection, mass measurement, and dynamic elastic modulus measurement), and then every 50 cycles thereafter. After the 200th cycle, the compressive strength of each specimen group was measured.

The mass loss, relative dynamic modulus of elasticity, and compressive strength reduction were calculated using Equations (1)–(3), respectively. The test results were averaged from the experimental data of all specimens to minimize the impact of random factors associated with individual specimens.

(1) $$W_{n}=(G_{0}-G_{n})/G_{0}$$

(2) $$P_{n}=f_{n}/f_{0}={T_{0}}^{2}/{T_{n}}^{2}$$

(3) $$f_{c}(n)=(f_{c,0}-f_{c,n})/f_{c,0}$$

where $W_{n}$ = the mass loss; $G_{0}$ and $G_{n}$ = the masses (in kg) of specimen after 0 and n cycles; $P_{n}$ = the relative dynamic modulus of elasticity after n cycles; $f_{0}$ and $f_{n}$ = the dynamic modulus of elasticity (in MPa) after 0 and n cycles; $T_{0}$ and $T_{n}$ = the natural vibration frequencies (in Hz) of specimen after 0 and n cycles; $f_{c}(n)$ = reduction in compressive strength; $f_{c,0}$ and $f_{c,n}$ = the compressive strengths (in MPa) of specimen after 0 and *n* cycles.

## 3. Analysis of Test Results

The primary effects of freeze–thaw cycles on concrete performance were reflected in mass loss, changes in relative dynamic elastic modulus, and compressive strength reduction. This section systematically analyzes the influence patterns of freeze–thaw cycles based on the performance of specimens with different strength grades.

### 3.1. Mass Loss Rate and Microstructural Characteristics

**Figure 6:**
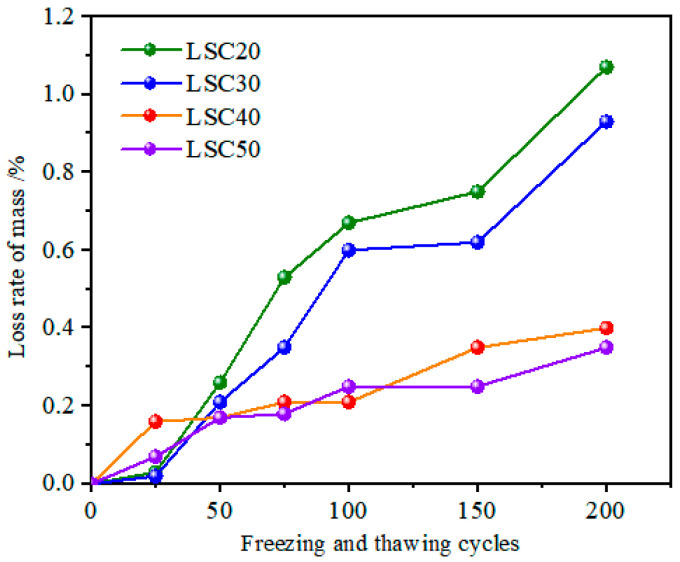
Mass loss rate of LSC at different strength grades under freeze–thaw cycles.

**Figure 7:**
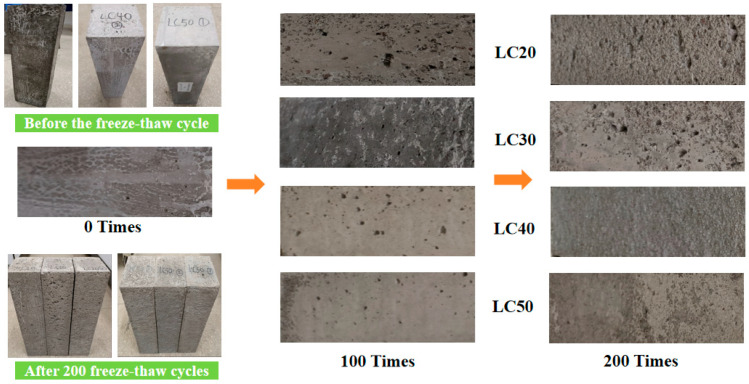
Appearance changes in LSC.

Figure 6 shows the curve of the mass loss rate of LSC at each strength grade with the number of freeze–thaw cycles. The mass loss rate of all specimens increased progressively with the number of freeze–thaw cycles, while higher-strength concretes exhibited lower mass loss rates. After 50 cycles, the differences among concretes of different strength grades were still insignificant, with the mass loss rates of LSC20, LSC30, LSC40, and LSC50 being 0.26%, 0.21%, 0.17%, and 0.17%, respectively. This is because, at the early stage of freeze–thaw action, the internal moisture of concrete is not yet fully saturated, and only a portion of the pore water freezes. Sufficient space remains for ice expansion, thereby limiting the stress induced by freezing and thawing. Consequently, the damage mainly manifests as slight surface cracking and local mortar scaling rather than through-cracks [31]. At this stage, the influence of pore size distribution and connectivity on freeze–thaw durability has not yet become significant, resulting in small differences in mass loss. With the further increase in freeze–thaw cycles, the mass loss rate of LSC20 and LSC30 specimens rose rapidly, whereas that of LSC40 and LSC50 specimens changed only slightly. After 200 cycles, the mass loss rates of LSC20, LSC30, LSC40, and LSC50 were 1.07%, 0.93%, 0.40%, and 0.35%, respectively. The surface appearance of concretes with different strength grades (Figure 7) was consistent with the trend in mass loss: concretes with higher strength exhibited better surface integrity after freeze–thaw cycling. Before the cycles, the specimen surfaces were smooth and dense; after 200 cycles, LSC20 and LSC30 showed increased pores, exposed aggregates, and severe scaling, whereas LSC40 and LSC50 exhibited only slight surface roughness and generally smooth surfaces. These findings further demonstrate that higher-strength concretes possess superior resistance to freeze–thaw deterioration. In low-strength concretes (LSC20 and LSC30), the higher water-to-cement ratio leads to higher porosity and stronger pore connectivity. After multiple freeze–thaw cycles, the near-surface regions gradually reach or even exceed the critical degree of saturation. When the pore water freezes, intensified ice crystallization and hydraulic pressure promote the propagation and coalescence of pre-existing microcracks, forming a cyclic amplification process of surface scaling, re-wetting, and re-freezing, which accelerates mass loss. In contrast, high-strength concretes (LSC40 and LSC50) possess denser pore structures, which limit water ingress and make it difficult to reach critical saturation. As a result, the internal pressure generated by freezing is limited, and the damage mainly manifests as slight surface mortar erosion, leading to minimal changes in mass loss [32].

**Figure 8:**
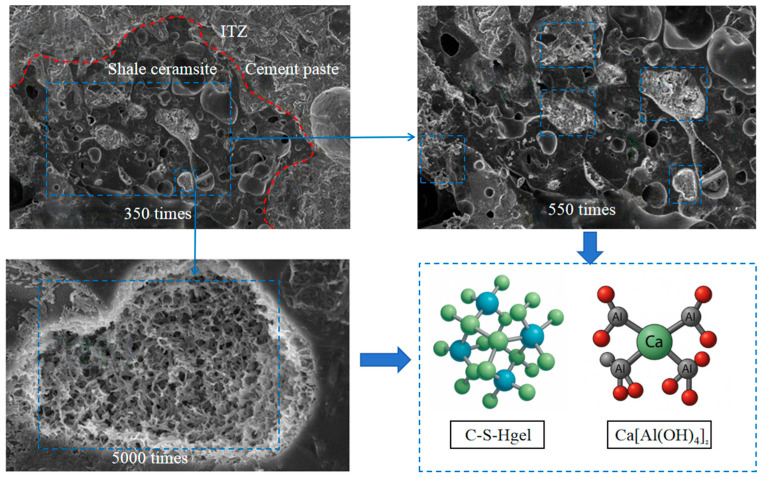
SEM images of LSC.

To further reveal the microstructural characteristics responsible for the observed freeze–thaw behavior, scanning electron microscopy (SEM) was used to examine the interfacial transition zone and hydration products of LSC. Figure 8 presents the scanning electron microscopy (SEM) images of LSC at various magnifications. The images show a dense interfacial transition zone (ITZ) between the ceramsite aggregate and the cement paste, with no visible microcracks or pore clustering, indicating strong interfacial bonding. At higher magnifications, abundant C–S–H gels and Ca[Al(OH)_4_]_2_ precipitates are observed, suggesting that the Al_2_O_3_ and SiO_2_ components in the ceramsite have undergone secondary reactions with the Ca(OH)_2_ from cement hydration. These reaction products fill the ITZ and ceramsite pore walls, reducing microvoids and improving interface density and stability. Unreacted white Ca(OH)_2_ crystals are still visible at the precipitate edges, confirming ongoing hydration and secondary reactions. These microstructural characteristics enhance ceramsite–cement matrix bonding, reduce moisture migration pathways, mitigate internal stress during freeze–thaw cycles, and ultimately improve frost resistance.

### 3.2. Mass Loss Rate and Appearance Changes

**Figure 9:**
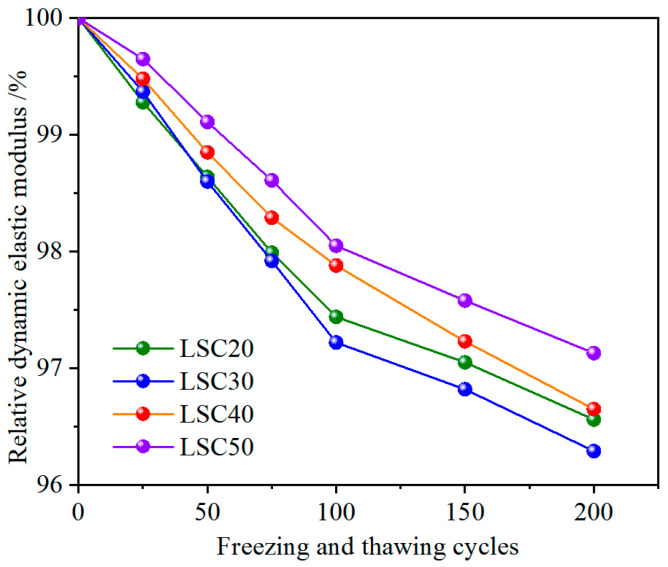
The variations in the relative dynamic elastic modulus of LSC.

Figure 9 illustrates the variations in the relative dynamic modulus of elasticity of LSC with different strength grades under freeze–thaw cycles. As the number of cycles increased, the relative dynamic modulus of elasticity of all specimens gradually decreased. Overall, the damage evolution of concrete under freeze–thaw cycles can be divided into two stages: an initial rapid deterioration stage and a subsequent gradual stabilization stage. During the first 100 cycles, the relative dynamic modulus of elasticity decreased rapidly due to the cyclic migration of water and the expansion–contraction of ice crystals, which generated considerable internal stresses and led to the rapid initiation and propagation of microcracks along the interfacial transition zone (ITZ) and pore boundaries [22]. For example, the relative dynamic modulus of elasticity of LSC20 and LSC50 specimens decreased by 2.56% and 1.95%, respectively, within the first 100 cycles. At this stage, the cracks were mainly fine and dispersed, corresponding to a notable reduction in the elastic stiffness of the material. In the subsequent 100 cycles, the pre-existing cracks gradually coalesced and released local stress concentrations, while the rate of new crack formation decreased, resulting in a slower decline in the relative dynamic modulus of elasticity. By the end of 200 freeze–thaw cycles, the total losses of LSC20 and LSC50 specimens were 3.44% and 2.87%, respectively. High-strength LSC (such as LSC40 and LSC50), with lower porosity and stronger aggregate–paste bonding, could effectively distribute localized freezing stresses and restrain crack propagation, thus maintaining a higher dynamic modulus of elasticity. In contrast, low-strength LSC (LSC20 and LSC30) exhibited a looser pore structure and more microdefects within the interfacial transition zone, where stress tended to concentrate during repeated freezing and thawing, leading to crack propagation along pores and interfaces and a more pronounced decrease in the dynamic modulus of elasticity.

### 3.3. Compressive Strength Losses

**Figure 10:**
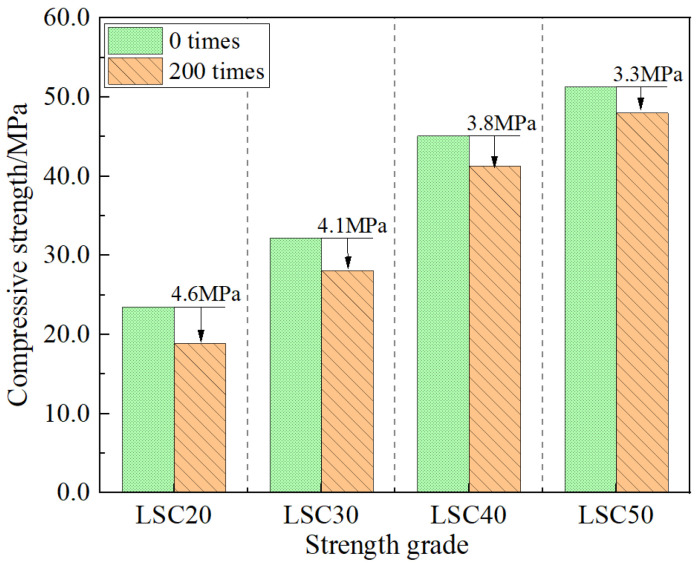
Impact of 200 freeze–thaw cycles on compressive strength.

Figure 10 presents the comparison of compressive strength of LSC before and after 200 freeze–thaw cycles. As shown, the compressive strength of all specimens decreased after 200 cycles, with higher-strength LSC exhibiting smaller reductions. The compressive strengths of LSC20, LSC30, LSC40, and LSC50 decreased by 4.6 MPa, 4.1 MPa, 3.8 MPa, and 3.3 MPa, corresponding to reductions of 19.57%, 13.77%, 9.21%, and 6.43%, respectively. Freeze–thaw cycling caused internal crack propagation and microstructural deterioration, thereby weakening the overall load-bearing capacity of the concrete. In low-strength concretes, microcracks tended to coalesce into interconnected fractures under repeated freezing and thawing, disrupting load-transfer paths and intensifying local stress concentrations, which in turn accelerated the degradation of compressive strength. Meanwhile, the weakened bonding between aggregates and the cement paste further reduced stress transmission efficiency, making the strength loss more pronounced. In contrast, high-strength concretes, characterized by a denser matrix and stronger interfacial adhesion, were able to maintain higher structural integrity under cyclic freezing stresses. Although microcracks were also present, they remained dispersed and non-penetrative, allowing the material to preserve better compressive stability and residual bearing capacity [33].

The changes in appearance, mass loss rate, relative dynamic elastic modulus, and compressive strength loss during freeze–thaw cycling exhibit a synchronous deterioration pattern, reflecting the same underlying degradation mechanisms. The increase in mass loss rate corresponds to surface spalling and pore exposure, which reduce the effective load-bearing cross section. This deterioration is accompanied by a decrease in relative dynamic elastic modulus, indicating internal microcrack propagation and degradation of the material’s elastic properties. These effects ultimately lead to a reduction in compressive strength, as both microstructural weakening and the decrease in relative dynamic elastic modulus diminish the material’s ability to resist external loads. Therefore, surface damage, mass loss, decrease in relative dynamic elastic modulus, and strength loss are intrinsically correlated and can jointly characterize the freeze–thaw damage evolution of LSC.

## 4. Probabilistic Parameter Estimation of Compressive Strength Degradation

### 4.1. Degradation Process of Compressive Strength Under Freeze–Thaw Cycle

The analysis of freeze–thaw cycle tests provides a preliminary understanding of the performance changes in LSC under freeze–thaw conditions. To further quantify the deterioration process, it is crucial to investigate the compressive strength degradation of LSC under freeze–thaw cycles. The dynamic elastic modulus, as a sensitive indicator of internal microstructural integrity, can effectively reflect the degree of material damage during freeze–thaw action. Therefore, in this study, the dynamic elastic modulus variation data of LSC20 to LSC50 specimens (as shown in Table 5) were selected and combined with compressive strength test results to establish a quantitative relationship between dynamic elastic modulus and compressive strength, providing a basis for characterizing the degradation behavior of LSC.

The functional relationship between the dynamic elastic modulus of ordinary concrete and the compressive strength of cubes was proposed in Ref. [34]. As reported in Ref. [35], ordinary concrete and LSC exhibit similar basic mechanical properties. Therefore, based on the functional relationship of ordinary concrete, a fitted relationship between the dynamic elastic modulus $E_{t}$ and the cube compressive strength of LSC $f_{cu}$ was established.

(4) $$E_{t}=38f_{cu}^{4/3}+13780$$

Ref. [35] constructed the relationship between the $f_{cu}$ of LSC and the axial compressive strength $f_{cd}$:

(5) $$f_{cd}={(f}_{cu}+0.46)/1.05$$

By substituting Equation (4) into Equation (5), the relationship between the axial compressive strength of the concrete and the dynamic elastic modulus can be seen at Equation (6).

(6) $$f_{cd}=\left[{\left(\frac{E_{t}-13780}{38}\right)}^{\frac{3}{4}}+0.46\right]/1.05$$

**Figure 11:**
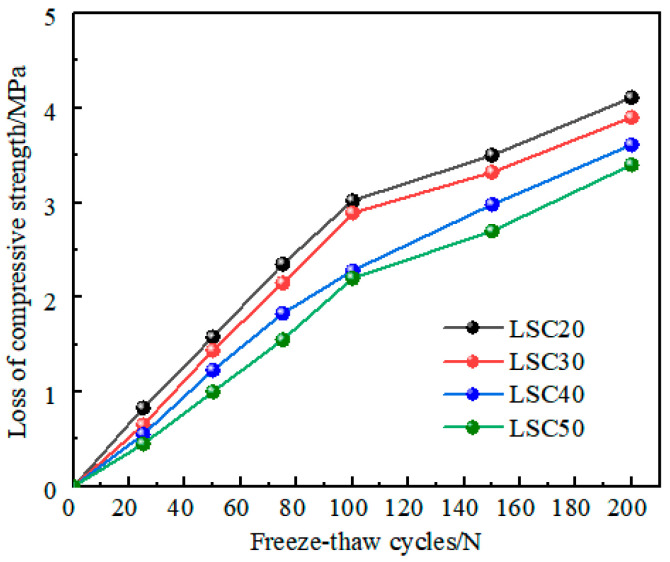
Time variation curve of compressive strength loss under freeze–thaw cycle.

According to Table 6, the degradation curves of compressive strength with freeze–thaw cycles for four grades of LSC (LSC20, LSC30, LSC40, and LSC50) were plotted. As shown in Figure 11, the figure illustrates that under the influence of freeze–thaw cycles, the compressive strength loss of concrete at all strength grades exhibits a monotonically increasing degradation trend, that is, the compressive strength of concrete decreases monotonically with the number of freeze–thaw cycles. Given the damage mechanism of concrete under freeze–thaw conditions and the applicability of the Gamma process in simulating monotonically decreasing degradation data, it can be reasonably inferred that the degradation process of concrete strength under freeze–thaw cycles approximately follows the Gamma process.

### 4.2. Definition and Applicability of the Gamma Procedure

To ensure that the compressive strength degradation of LSC complies with the Gamma process {G(N),N≥0} with shape parameter α(N) and rate parameter β, the following three conditions must be met:

(1) Initial condition: When the number of freeze–thaw cycles N=0, the degradation value is 0.

(2) Independent increment: For any $N_{1}<N_{2}<\cdots N_{n}$, Increments $G\left(N_{2}\right)-G\left(N_{1}\right)$, $G\left(N_{3}\right)-G\left(N_{2}\right)$, …$G(N_{n})-G(N_{n-1})$ are mutually independent.

(3) Gamma distribution: For any N>s, increment G(N)−G(s) follows the Gamma distribution with shape parameter α(N)−α(s) and rate parameter β.

The probability density function of Gamma distribution increment is [36]:

(7) $$f(x,\alpha (N),\beta )=\frac{\beta^{\alpha (N)}}{\Gamma (\alpha (N))}x^{\alpha (N)-1}e^{-\beta x},x\geq 0$$

It should be noted that when the shape parameter $\alpha \left(N\right)<1$, the probability density function of the Gamma distribution tends to infinity as x→0, implying a theoretical domain of x>0. However, in engineering applications such as modeling the degradation of compressive strength in LSC, degradation is typically non-negative and often begins from zero. Therefore, the domain is practically extended to x≥0 in Equation (7) to accommodate the initial undegraded state (N=0). This adjustment does not affect the validity of the model, as the probability of observing zero degradation at N>0 remains negligible.

To ensure the numerical stability and identifiability of parameter estimation, a nondimensionalization of the freeze–thaw cycle number was applied in this study. Let $N_{max}$ denote the maximum number of freeze–thaw cycles, and define the normalized variable as follows:

(8) $$n=\frac{N}{N_{max}}$$

where $N_{max}$ denotes the maximum number of freeze–thaw cycles for the given test group (200 in this study). The normalized shape parameter can then be expressed as:

(9) $$\alpha \left(n\right)=an^{b}$$

This normalization only affects the optimization process, while the physical meaning of the model remains unchanged. All fitting results and predicted values are calculated and plotted based on the actual number of freeze–thaw cycles; therefore, the obtained model still accurately reflects the compressive strength degradation behavior of LSC under real freeze–thaw conditions.

Therefore, the probability density function of the normalized Gamma distribution can be expressed as:

(10) $$f(x,\alpha (n),\beta )=\frac{\beta^{\alpha (n)}}{\Gamma (\alpha (n))}x^{\alpha (n)-1}e^{-\beta x},x\geq 0$$

where α(n) is the shape parameter, representing the rate or cumulative effect of degradation and β is the rate parameter, characterizing the scale or variability of the degradation magnitude. Γ(α(n)) denotes the Gamma function, which is defined as:

(11) $$\Gamma (\alpha (n))=\int_{0}^{\infty }x^{\alpha -1}e^{-x}dx$$

**Figure 12:**
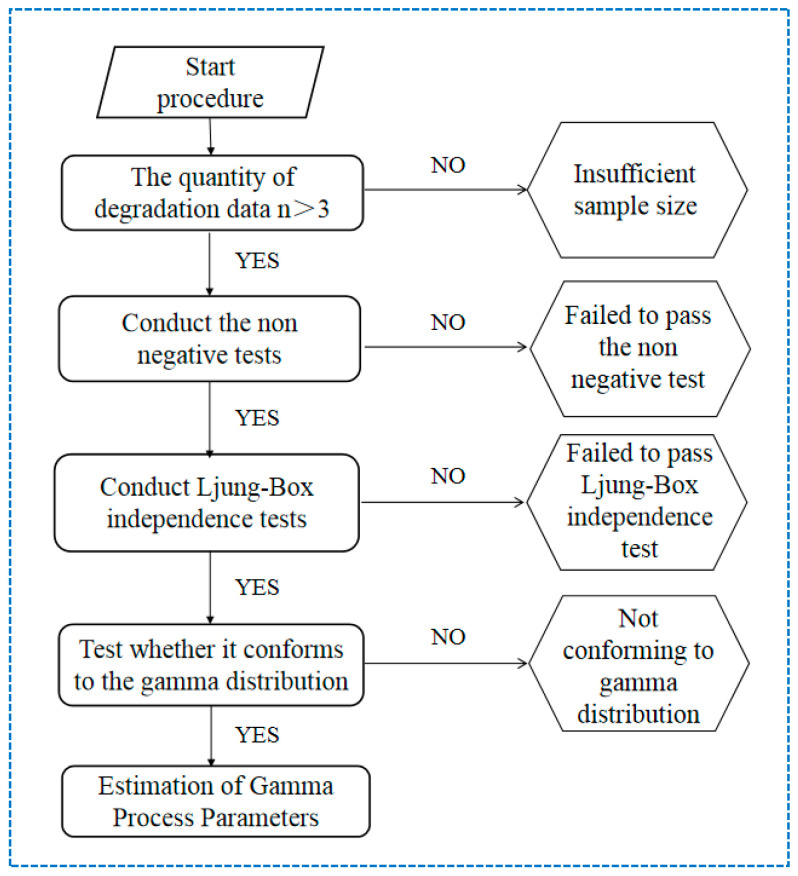
Gamma process identification and parameter estimation flowchart.

The strength degradation of LSC begins at N=0, satisfying the first condition. For the second and third conditions, the MATLAB2021A program is prepared to test the degradation data, and the specific process is shown in Figure 12:

The cumulative compressive strength degradation values of LSC in Table 5 were input into the program, which directly performed the Ljung–Box independence test on the degradation data. The results are displayed in Table 7. It can be observed that after conducting the Ljung–Box test with one to three lags, all *p*-values exceeded the given significance level of 0.05, indicating that the degradation values at different freeze–thaw cycles are independent of each other. This validates the hypothesis of statistical independence for the degradation data.

A Kolmogorov–Smirnov (K–S) test was conducted to verify whether the compressive strength degradation values follow the Gamma distribution required for the Gamma process model. The data from Table 6 were input into the program with a confidence level of 95%, and the results are presented in Table 8. The results indicate that the compressive strength degradation values for LSC20, LSC30, LSC40, and LSC50 all fit the Gamma distribution well. The *p*-values for all samples are greater than 0.90, exceeding the significance level of 0.05. This confirms that the degradation values follow the Gamma distribution, satisfying the statistical assumption of the Gamma process model.

### 4.3. Gamma Process-Based Model for the Compressive Strength Degradation of LSC

The compressive strength degradation of LSC is described using a power–law relationship and a Gamma distribution model. This model assumes that the degradation value X(N) gradually accumulates as the number of freeze–thaw cycles *N* increases, and on this basis, the Gamma distribution is employed to represent the uncertainty in the degradation process. It is assumed that the degradation value X(N) at the *N*th freeze–thaw cycle satisfies the power–law relationship:

(12) $$X(N)=a\cdot N^{b}$$

where a = the initial degradation rate and *b* = the power–law exponent describing the rate of degradation. To estimate the parameter b, we use a logarithmic transformation to simplify the data processing:

(13) $$logX\left(N\right)=loga+blogN$$

This equation suggests that, in the logarithmic space, the degradation value $X\left(N\right)$ and the number of freeze–thaw cycles N exhibit a linear relationship. Therefore, the parameters β and a can be estimated through linear regression. Based on the least-squares method, the estimation formula for the power–law exponent *b* is given as [37]:

(14) $$b=\frac{\sum_{i=1}^{n}log(\frac{N_{i}}{N_{n}})log(\frac{X_{i}}{X_{n}})}{\sum_{i=1}^{n}log{\left(\frac{N_{i}}{N_{n}}\right)}^{2}}$$

To further describe the volatility in the degradation process, in the Gamma process modeling stage, the aforementioned normalized variable *n* = *N*/*N_max_* was introduced in this paper. Here, *N_max_* was set to 200, which represents the maximum number of freeze–thaw cycles in the test. Normalization is only to improve the stability of numerical calculations and does not affect the physical meaning of the power–law relationship $X(N)=a\cdot N^{b}$.

Therefore, it is assumed that the degradation value X(N) follows a Gamma distribution in each freeze–thaw cycle stage:

(15) $$X\left(N\right)\sim Gamma\left(\alpha \left(N\right),\beta \right),\;\alpha \left(N\right)=a{\left(\frac{N}{N_{max}}\right)}^{b}\;N_{max}=200$$

where the shape parameter ${\alpha \left(N\right)=a(N/N_{max})}^{b}$ changes with *N*, reflecting the expected value of the degradation, while the rate parameter β controls the overall rate of change and the fluctuation amplitude of the degradation process. By introducing a gamma distribution, the model can describe the random fluctuations of the actual degradation around the expected value of the power–law relationship, thus more accurately reflecting the degradation behavior of materials in complex environments. Specifically, the Gamma distribution’s probability density function is:

(16) $$f_{X|N}(x|N)=\frac{\beta^{\alpha \left(N\right)}x^{\alpha \left(N\right)-1}e^{-\beta x}}{\Gamma [\alpha \left(N\right)]}$$

Among them:

(17) $${\alpha \left(N\right)=a(N/200)}^{b},x>0$$

Given *n* observed freeze–thaw cycles $N_{1},N_{2},\ldots N_{n}$, the degradation values $X(N_{1}),X(N_{2}),\ldots ,X(N_{n})$ at each cycle also follow a Gamma distribution. Therefore, the likelihood function can be expressed as:

(18) $$L(a,\beta )=\prod_{i=1}^{n}\frac{\beta^{\alpha \left(N_{i}\right)}{X\left(N_{i}\right)}^{\alpha \left(N_{i}\right)-1}e^{-\beta X(N_{i})}}{\Gamma [\alpha \left(N_{i}\right)]}$$

The logarithm is applied to the likelihood function to derive the log-likelihood function:

(19) $$logL(a,\beta )=\sum_{i=1}^{n}\left[\alpha \left(N_{i}\right)log(\beta )+(\alpha \left(N_{i}\right)-1)logX\left(N_{i}\right)-\beta X\left(N_{i}\right)-log\Gamma [\alpha \left(N_{i}\right)]\right]$$

To find the optimal values of *a* and β, partial derivatives with respect to *a* and β are taken and set to zero. The partial derivative with respect to *a* is given as:

(20) $$\frac{\partial logL}{\partial a}=\sum_{i=1}^{n}{\left(\frac{N_{i}}{200}\right)}^{b}[log\beta +log\left(X\left(N_{i}\right)\right)-\psi \left(\alpha \left(N_{i}\right)\right)]=0$$

where

(21) $$\psi (\alpha \left(N_{i}\right))=\frac{d}{d\alpha \left(N_{i}\right)}log\Gamma (\alpha \left(N_{i}\right))$$

The partial derivative with respect to β is given as:

(22) $$\frac{\partial logL}{\partial \beta }=\sum_{i=1}^{n}[\frac{\alpha \left(N_{i}\right)}{\beta }-X(N_{i})]=0$$

In the actual calculation, the power–law exponent *b* is first estimated preliminarily according to Equation (14) to enhance the convergence stability of parameter identification. Subsequently, *b* is substituted into the joint likelihood equations constructed by Equations (20) and (22), and the optimal estimates of parameters a and *β* are obtained through numerical iteration. Here, $\psi (\alpha \left(N_{i}\right))$ denotes the Digamma function, which has no analytical solution and thus requires numerical optimization for computation.

According to the properties of the Gamma distribution, the expected value and standard deviation of the degradation quantity can be calculated respectively using Equations (23) and (24):

(23) $$E\left[X(N)\right]=\frac{\alpha \left(N\right)}{\beta }$$

(24) $$\sigma_{X(N)}=\frac{\sqrt{\alpha \left(N\right)}}{\beta }$$

The shape parameter of the degradation value $a{\left(\frac{N}{N_{max}}\right)}^{b}$ increases with the number of freeze–thaw cycles *N*. When the shape parameter becomes large, the probability density curve of the Gamma distribution gradually approaches symmetry and can be approximated by a normal distribution $\left[N(\mu ,\sigma^{2})\right]$ according to the central limit theorem [38]. Therefore, in the subsequent model validation stage, the normal approximation method is employed to construct the confidence interval of the degradation value. At a 95% confidence level, the standard normal quantile is $z_{0.975}=1.96$, and the confidence interval can be expressed as:

(25) $${CI}_{95\%}=E[X(N)\pm 1.96\sigma_{X(N)}]$$

To verify the reliability of the Gamma process degradation model, three indicators—the coefficient of determination ($R^{2}$), root mean square error (RMSE), and mean absolute percentage error (MAPE)—were employed to quantitatively evaluate the fitting accuracy.

(26) $$R^{2}=1-\frac{\sum_{I=1}^{n}{\left(X_{i}-\hat{X_{i}}\right)}^{2}}{\sum_{I=1}^{n}{\left(X_{i}-\bar{X}\right)}^{2}}$$

(27) $$RMSE=\sqrt{\frac{1}{n}\sum_{i=1}^{n}{\left(X_{i}-\hat{X_{i}}\right)}^{2}}$$

(28) $$MAPE=\frac{100}{n}\sum_{i=1}^{n}\left|\frac{X_{i}-\hat{X_{i}}}{X_{i}}\right|$$

where $X_{i}$ is the measured degradation value, $\bar{X}$ is the mean of the measured degradation values, and $\hat{X_{i}}$ is the predicted mean value from the model.

### 4.4. Degradation Modeling Results and Analysis

For four different strength levels of LSC (LSC20, LSC30, LSC40, LSC50), the compressive strength degradation characteristics were modeled using the Gamma distribution. Through fitting the experimental data, the corresponding shape parameters, rate parameters, and ratio coefficients for each strength level were precisely determined. The specific parameters are presented in Table 8. From an engineering perspective, the empirical equations in Table 9 are not merely mathematical fitting functions, because their parameters directly reflect the freeze–thaw degradation characteristics of LSC. Specifically, parameter *a* represents the initial or reference performance level of the material and determines the overall level of the degradation curve; parameter *b* characterizes the sensitivity of strength deterioration to increasing freeze–thaw cycles, with a larger value generally indicating a slower degradation rate and thus better frost resistance; and parameter β reflects the dispersion and stability of the degradation process, which is associated with the heterogeneity of pore structure, interfacial transition zone quality, and crack development. Therefore, the proposed degradation models not only provide mathematical fitting of the experimental data but also offer a practical tool for predicting the long-term mechanical performance of LSC in cold-region engineering applications.

To verify the accuracy and reliability of the Compressive strength degradation model for the LSC, an error analysis was conducted. The results are shown in Table 10. It can be seen that the $R^{2}$ values for all four strength grades are greater than 0.96, the *RMSE* values are less than 0.25 MPa, and the *MAPE* is controlled within the range of 8–11%. This indicates that the Gamma process degradation model has reliable prediction capabilities and strong robustness.

**Figure 13:**
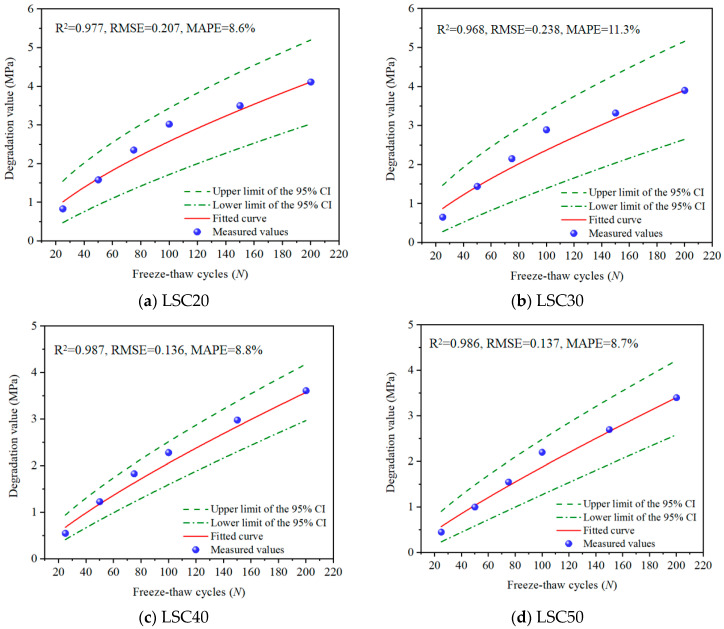
Comparison of Gamma process-fitted degradation curves and measured values for LSC.

The compressive strength degradation curve of the LSC was fitted and compared with the average degradation values of the measured compressive strength for each specimen group, as shown in Figure 13. Each plotted data point represents the mean of ten parallel tests. Although only the mean values are displayed, all individual degradation data (a total of 60 data points) calculated from Equation (6) fell entirely within their corresponding 95% confidence intervals. This indicates that the model effectively envelopes the experimental data fluctuations and adequately captures the stochastic characteristics of the cumulative freeze–thaw damage process in LSC. Meanwhile, the fitted curves exhibit a high degree of consistency with the experimental trends, further confirming the accuracy, stability, and applicability of the Gamma process model in characterizing the freeze–thaw degradation behavior of LSC.

**Figure 14:**
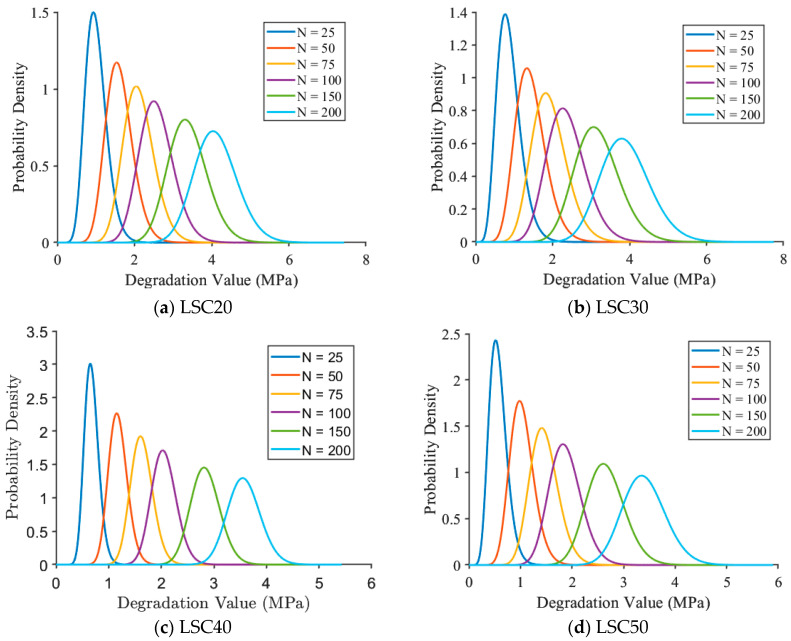
Probability density function of compressive strength degradation of LSC of different grades.

Figure 14 illustrates the probability density curves of compressive strength degradation for LSC with different strength grades. For LSC20 and LSC30, the probability density curves of degradation exhibit a pronounced right-skewed distribution at different freeze–thaw cycles. In the early stage of freeze–thaw exposure (N ≤ 50), the probability density functions show high peaks at low degradation values, indicating that damage mainly occurs within a mild degradation range and that the system remains in a slow deterioration phase. As the number of freeze–thaw cycles increases (N=75–150), the peaks of the curves gradually decrease and shift rightward, accompanied by a significant widening of the distribution range. This reflects an increase in the randomness of degradation, as the cumulative propagation of internal microcracks and interfacial damage accelerates the loss of compressive strength. When the number of freeze–thaw cycles further increases to 200, the probability density curves become flatter with elongated tails, suggesting greater uncertainty and spatial nonuniformity in the degradation process. In contrast, the probability density curves of LSC40 and LSC50 (Figure 13c,d) are generally flatter, with lower peaks and broader distributions. At the early stage (N≤50), the degradation values of both remain concentrated within a narrow range, implying that high-strength concretes possess stronger resistance to early freeze–thaw deterioration. As the number of cycles increases N=75–200, the distributions gradually broaden and the peaks decrease, but the rightward shift is limited, indicating a more uniform and slower degradation process. This behavior reflects that the denser pore structure and more stable interfacial transition zone of high-strength LSC effectively suppress the propagation of microcracks and delay the degradation of compressive strength under repeated freeze–thaw action.

**Figure 15:**
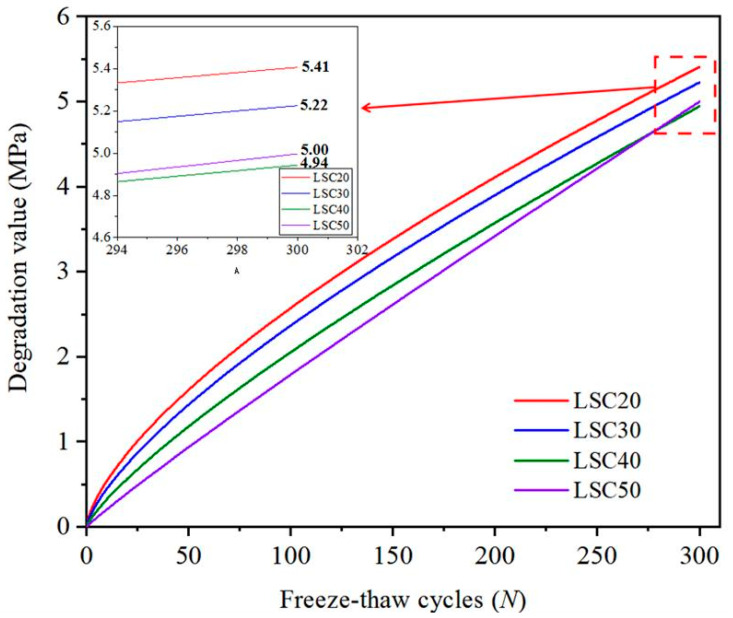
The degradation curves of different strength grades of LSC.

Figure 15 illustrates the degradation curves of different strength grades of concrete under freeze–thaw cycles, along with the extrapolated degradation trends predicted up to 300 cycles based on the Gamma process model. As shown in the figure, the degradation curves for all concrete grades increase progressively with the number of freeze–thaw cycles, and the degradation rate of high-strength LSC (such as LSC40 and LSC50) is significantly lower than that of low-strength LSC (such as LSC20). At 300 freeze–thaw cycles, the extrapolated degradation values are 5.41 MPa for LSC20, 5.22 MPa for LSC30, 5.00 MPa for LSC50, and 4.94 MPa for LSC40. The slightly higher degradation value of LSC50 compared with LSC40 lies within the 95% confidence interval, reflecting the uncertainty associated with model extrapolation rather than a change in the intrinsic material properties. Due to their denser pore structures and more brittle interfacial transition zones, high-strength LSC may experience greater internal crack propagation under freeze–thaw exposure, leading to a degradation rate that approaches or even slightly exceeds that of medium-strength LSC in the later stages [39]. Therefore, although this phenomenon is physically reasonable to some extent, further long-term experiments are still required to verify the accuracy of the extrapolated predictions.

This study compared the compressive strength degradation behavior of LSC with that of ordinary concrete (OC) reported in Ref. [40] under freeze–thaw conditions. As shown in Table 11, it can be observed that, at the same number of freeze–thaw cycles, the strength loss of OC is consistently and significantly higher than that of LSC, and the difference between them gradually increases with the number of cycles. After 200 freeze–thaw cycles, the strength losses of C30, C40, and C50 reached 11.94, 10.05, and 11.48 MPa, respectively, whereas the corresponding LSC30, LSC40, and LSC50 concretes exhibited losses of only 3.90, 3.57, and 3.42 MPa—representing 32.66%, 35.52%, and 29.79% of the respective values for OC. This indicates that LSC has a lower degradation rate and more stable mechanical performance under freeze–thaw exposure. The superior performance of LSC primarily originates from differences in pore structure and interfacial characteristics between the two materials. The lightweight shale aggregate in LSC possesses a composite pore structure with a dense outer shell and an internally porous core, in which some pores are partially closed. This configuration allows the aggregate to absorb and release the volumetric expansion energy generated during freezing and thawing, acting as an internal stress buffer that reduces pore pressure concentration. Meanwhile, the ITZ between lightweight aggregate and cement paste is more homogeneous and compact, lowering stress concentrations and hindering the coalescence of microcracks. In contrast, ordinary concrete exhibits a more porous ITZ and a greater number of microcrack pathways, making it more susceptible to internal deterioration caused by ice crystal growth. These findings confirm the superior freeze–thaw durability and long-term applicability of LSC in structural engineering.

## 5. Results and Discussion

Although the proposed Gamma process-based model provides an effective framework for describing the compressive strength degradation of LSC under freeze–thaw cycles, several limitations should be noted. First, the model parameters were calibrated based on the experimental data obtained in this study, and their applicability to other types of lightweight concrete or different environmental conditions requires further validation. Second, the degradation process was assumed to follow a stochastic Gamma process, which may simplify the complex physical mechanisms involved in freeze–thaw damage. Future research could extend the model by incorporating multi-factor environmental effects and larger datasets to further improve its general applicability.

## 6. Conclusions

This study systematically investigates the freeze–thaw resistance of LSC through basic mechanical property tests and freeze–thaw cycle experiments. A compressive strength degradation model for LSC under freeze–thaw conditions is developed. The main conclusions of this study are as follows:

(1) With increasing freeze–thaw cycles, the mass, dynamic modulus, and compressive strength of LSC gradually decreased. High-strength LSC showed markedly better frost resistance than low-strength LSC. After 200 cycles, the mass loss rate, relative dynamic modulus reduction, and compressive strength loss of LSC50 were only 0.35%, 2.87%, and 6.43%, respectively, which were significantly lower than those of LSC20 (1.07%, 3.44%, and 19.57%). The denser pore structure and stronger aggregate–paste bonding of high-strength LSC effectively mitigate internal cracking and freeze–thaw damage, ensuring superior durability under cyclic freezing and thawing.

(2) The experimental results show that the compressive strength degradation of LSC under freeze–thaw action follows a cumulative evolution trend and is strongly dependent on strength grade. This indicates that freeze–thaw deterioration of LSC cannot be fully characterized by a single stiffness-related indicator alone, and that compressive strength should be considered together with the relative dynamic elastic modulus in freeze–thaw deterioration assessment.

(3) The proposed Gamma process-based stochastic model showed good agreement with the experimental data for different strength grades of LSC, indicating that it is capable of describing the cumulative and random characteristics of compressive strength degradation under repeated freeze–thaw cycles, thereby providing a probabilistic basis for durability evaluation and long-term performance assessment of LSC under freeze–thaw conditions.

## Figures and Tables

**Table 1:** Major chemical compositions of the materials.

Material	SiO_2_	Al_2_O_3_	Fe_2_O_3_	K_2_O	MgO	SO_3_	CaO
Shale ceramic	63.5%	18.3%	7.3%	3.7%	2.2%	0%	5.0%
Shale pottery sand	63.5%	18.3%	7.3%	3.7%	2.2%	0%	5.0%
Ball-milled powder	55.0%	26.0%	7.0%	0%	2.0%	1.0%	9.0%
P.C 42.5 common Portland cement	17.8%	4.2%	5.7%	0.9%	1.7%	2.3%	67.4%
P.O 52.5 common Portland cement	20.7%	3.9%	2.9%	0.6%	1.9%	2.5%	67.5%

**Table 2:** Mix ratios of LSC (kg/m^3^ and mass percentage).

Strength Grade	Cement	Shale Ceramic	Shale Pottery Sand	Ball-Milled Powder	Admixture	Water	Unit Weight
LSC20	420 kg/m^3^ (28.6%)	290 kg/m^3^ (19.7%)	410 kg/m^3^ (27.9%)	90 kg/m^3^ (6.1%)	10.2 kg/m^3^ (0.7%)	250 kg/m^3^ (17.0%)	14.65 kg/m^3^
LSC30	460 kg/m^3^ (30.3%)	280 kg/m^3^ (18.5%)	430 kg/m^3^ (28.4%)	90 kg/m^3^ (5.9%)	11.0 kg/m^3^ (0.7%)	245 kg/m^3^ (16.2%)	15.10 kg/m^3^
LSC40	490 kg/m^3^ (29.6%)	320 kg/m^3^ (19.3%)	500 kg/m^3^ (30.2%)	110 kg/m^3^ (6.7%)	15.0 kg/m^3^ (0.9%)	220 kg/m^3^ (13.3%)	15.90 kg/m^3^
LSC50	520 kg/m^3^ (30.3%)	310 kg/m^3^ (18.1%)	540 kg/m^3^ (31.5%)	110 kg/m^3^ (6.4%)	15.8 kg/m^3^ (0.9%)	220 kg/m^3^ (12.8%)	16.46 kg/m^3^

**Table 3:** Mechanical properties.

Strength Grade	Cube Compressive Strength (MPa)				Mass (kg)	Axial Compressive Strength (MPa)	Dynamic Elastic Modulus (MPa)	Static Elastic Modulus (MPa)	Natural Frequency (Hz)
	3 d	7 d	14 d	28 d					
LSC20	12.7	13.8	18.4	23.5	5.937	23.3	1.533 × 10^4^	1.300 × 10^4^	1756
LSC30	18.5	22.2	26.3	32.2	6.082	31.1	1.669 × 10^4^	1.400 × 10^4^	1976
LSC40	27.8	32.0	36.7	45.1	6.511	42.3	2.115 × 10^4^	1.790 × 10^4^	1942
LSC50	32.9	37.8	42.5	51.3	6.742	50.3	2.253 × 10^4^	1.850 × 10^4^	1979

**Table 4:** Statistical parameters of 28-day Cube strength for LSC.

Strength Grade	Mean Value (MPa)	Maximum Value (MPa)	Minimum Value (MPa)	Standard Deviation (MPa)	Coefficient of Variation (%)
LSC20	23.5	24.4	22.5	0.71	3.00
LSC30	32.2	33.4	31.1	0.83	2.57
LSC40	45.1	45.7	44.2	0.57	1.27
LSC50	51.3	52.2	50.7	0.53	1.02

**Table 5:** Effect of freeze–thaw cycles on the dynamic elastic modulus of LSC.

Number of Freeze–Thaw Cycles	Dynamic Modulus of Elasticity/MPa			
	LSC20	LSC30	LSC40	LSC50
0	15,332	16,692	21,159	22,531
25	15,221	16,586	21,050	22,437
50	15,123	16,459	20,916	22,324
75	15,024	16,345	20,797	22,211
100	14,940	16,229	20,710	22,078
150	14,880	16,162	20,573	21,975
200	14,805	16,072	20,450	21,833

**Table 6:** The change of concrete compressive strength loss with time.

Number of Freeze–Thaw Cycles	Compressive Strength Loss/MPa			
	LSC20	LSC30	LSC40	LSC50
0	0	0	0	0
25	0.83	0.65	0.55	0.45
50	1.58	1.44	1.23	1.00
75	2.35	2.15	1.83	1.55
100	3.02	2.89	2.28	2.20
150	3.50	3.32	2.98	2.70
200	4.11	3.90	3.61	3.40

**Table 7:** Ljung–Box test *p*-values for each group.

Lags	LSC20	LSC30	LSC40	LSC50
First-order.	0.063	0.058	0.067	0.064
Second-order.	0.150	0.135	0.159	0.145
Third-order.	0.246	0.221	0.269	0.239

**Table 8:** The K-S test results.

Type of Concrete	LSC20	LSC30	LSC40	LSC50
*p*-Value	0.95	0.92	0.99	0.97

**Table 9:** Compressive strength degradation model and parameters of LSC with different strength grades.

Strength Level	a	*b*	β	Compressive Strength Degradation Model
LSC20	55.086	0.676	13.403	$f(X\left(N\right))=\frac{13.403^{55.086{\times (\frac{N}{200})}^{0.676}}{X(\frac{N}{200})}^{\left[55.086\times {\left(\frac{N}{200}\right)}^{0.676}-1\right]}e^{-13.403\times (\frac{N}{200})}}{\Gamma [55.086\times {\left(\frac{N}{200}\right)}^{0.676}]}$
LSC30	37.003	0.721	9.487	$f(X\left(N\right))=\frac{9.487^{37.003{\times (\frac{N}{200})}^{0.721}}{X(\frac{N}{200})}^{\left[37.003\times {\left(\frac{N}{200}\right)}^{0.721}-1\right]}e^{-9.487\times (\frac{N}{200})}}{\Gamma [37.003\times {\left(\frac{N}{200}\right)}^{0.721}]}$
LSC40	133.463	0.800	37.344	$f(X\left(N\right))=\frac{37.344^{133.463{\times (\frac{N}{200})}^{0.800}}{X(\frac{N}{200})}^{\left[133.463\times {\left(\frac{N}{200}\right)}^{0.800}-1\right]}e^{-37.344\times (\frac{N}{200})}}{\Gamma [133.463\times {\left(\frac{N}{200}\right)}^{0.800}]}$
LSC50	119.121	0.935	34.831	$f(X\left(N\right))=\frac{34.831^{119.120{\times (\frac{N}{200})}^{0.935}}{X(\frac{N}{200})}^{\left[119.120\times {\left(\frac{N}{200}\right)}^{0.935}-1\right]}e^{-34.831\times (\frac{N}{200})}}{\Gamma [119.120\times {\left(\frac{N}{200}\right)}^{0.935}]}$

Note: a represents the ratio coefficient, *b* represents the power–law exponent, and β represents the rate parameter.

**Table 10:** Goodness-of-fit statistics for the compressive strength degradation model of LSC with different strength grades.

Strength Level	$R^{2}$	*RMSE*	*MAPE*
LSC20	0.977	0.207	8.6%
LSC30	0.968	0.238	11.3%
LSC40	0.987	0.136	8.8%
LSC50	0.986	0.137	8.7%

Note: $R^{2}$ represents the coefficient of determination, *RMSE* represents the root mean square error, MAPE represents the mean absolute percentage error.

**Table 11:** Comparison of compressive strength loss in OC and LSC.

Time/*N*	Compressive Strength Loss/MPa					
	C30	LSC30	C40	LSC40	C50	LSC50
0	0	0	0	0	0	0
50	4.27	1.44	6.80	1.18	2.26	0.94
100	7.37	2.37	6.21	2.05	6.45	1.79
150	9.50	3.17	7.65	2.84	8.61	2.61
200	11.94	3.90	10.05	3.57	11.48	3.42

## Data Availability

The original contributions presented in this study are included in the article. Further inquiries can be directed to the corresponding author.

## Abbreviations

The following abbreviations are used in this manuscript:

LSC	Shale-based Lightweight Structural Concrete
OC	Ordinary Concrete
$R^{2}$	The Coefficient of Determination
*RMSE*	Root Mean Square Error
*MAPE*	Mean Absolute Percentage Error
a	The Ratio Coefficient
*b*	The Power–law Exponent
β	The Rate Parameter
